# Cholesteatoma of the Sphenoid Wing

**DOI:** 10.7759/cureus.88025

**Published:** 2025-07-15

**Authors:** Crina M Peterson, Shiwei Huang, Liam Chen, Walter Galicich

**Affiliations:** 1 Neurosurgery, University of Minnesota, Minneapolis, USA; 2 Laboratory Medicine and Pathology, University of Minnesota, Minneapolis, USA; 3 Neurosurgery, Hennepin County Medical Center, Minneapolis, USA

**Keywords:** bony destruction, inflamed cholesteatoma, mass effect, proptosis, sphenoid wing

## Abstract

Intracranial cholesteatoma is a rare entity most commonly present in the middle ear region. Due to this, a consensus on serial imaging and when to intervene is not well established. Here, we present a rare case of an HIV-positive patient without any prior history of trauma or infection who developed a T1 and T2 hyperintense lesion of the sphenoid wing, confirmed as cholesteatoma, that nearly doubled in size over six years, causing mass effect on the left orbit and ultimately requiring resection. To the best of our knowledge, this is the first reported case of sphenoid wing cholesteatoma without sinonasal/middle ear involvement in an HIV positive patient. Overall, we recommend that these lesions be followed with interval imaging and offered resection when rapid growth or mass effect is observed.

## Introduction

Cholesteatoma is a well-demarcated cystic lesion characterized by a squamous epithelium capsule filled with keratin debris. Although it is noncancerous, cholesteatoma can be locally invasive and cause bony destruction and/or remodeling [1]. Acquired cholesteatoma occurs either as a result of retraction of keratin epithelium from low middle ear pressure with an intact eardrum or migration of keratin epithelium through a perforated eardrum from infection, trauma, or surgery [1,2]. In contrast, congenital cholesteatomas typically present during childhood without prior history of infection or surgery, arising from trapped epithelial cell crests behind an intact eardrum during middle ear development [1-4]. Congenital cholesteatomas, also referred to as epidermoids, can be further divided into intracranial epidermoids and epidermoids of the diploe, with intradiploic epidermoid cysts being very uncommon but rarely reported in flat bones of the calvarium, including the sphenoid bone [5]. Although there have been reported cases of sphenoid sinus cholesteatoma and intracranial cholesteatoma after chronic otitis media, we present a rare case of adult cholesteatoma at the sphenoid wing without sinonasal or middle ear involvement and highlight diagnostic challenges in atypical locations [2-4].

## Case presentation

**Figure 1:**
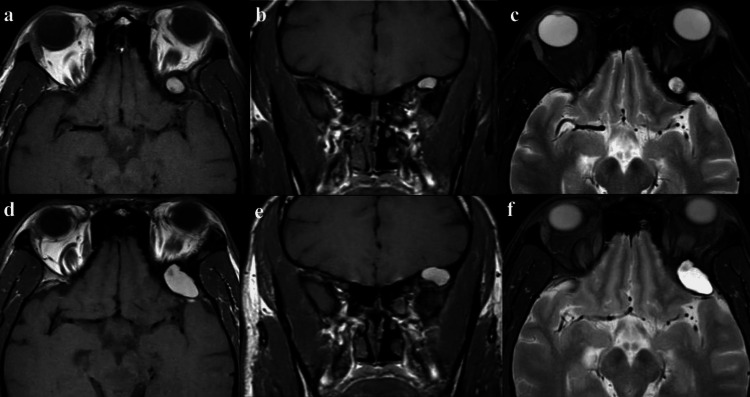
Left sphenoid wing lesion with mass effect (a, b) Axial and coronal T1-weighted MR images showing an 11 x 7 mm expansile, heterogeneous, hyperintense lesion in the left greater wing of the sphenoid, causing mass effect in the left orbital content. (c) Axial T2-weighted MR image showing hyperintense signal changes. (d-f) Axial and coronal T1-weighted MR images and axial T2-weighted MR demonstrating interval growth in the lesion to 24 x 15 mm over six years.

A 39-year-old male with a history of HIV on antiretroviral treatment presented for an elective resection of a left sphenoid wing mass that nearly doubled in size over six years. The mass was initially discovered during a workup of left proptosis. On examination, the patient only had mild lateral gaze paresis in the left eye that occasionally caused diplopia. MRI was performed, which showed a heterogenous T1 and T2 hyperintense lesion in the left sphenoid wing, causing mass effect on the orbital content (Figures 1a-1c). This lesion did not contrast-enhance and did not demonstrate diffusion restriction (not shown).

**Figure 2:**
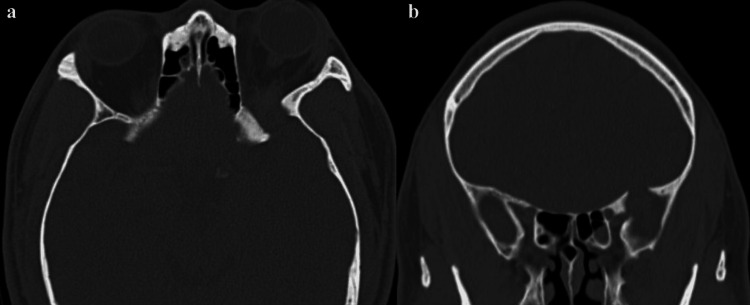
Bony effects of the left sphenoid wing lesion Figure 2 demonstrates CT bony windows, both axial (a) and coronal (b) that illustrate bony remodeling in the sphenoid wing and lateral orbital wall.

CT showed bony remodeling in the sphenoid wing and lateral orbital wall (Figure 2).

The patient was initially managed with serial MRI images; however, prior to the elective procedure, he reported worsening proptosis with subsequent MRI demonstrating growth of the lesion to approximately double in size (Figures 1d-1f). Given the growth of the lesion and the patient’s worsening symptoms, the surgery was offered to remove the lesion and establish a diagnosis.

A left anterior/middle fossa skull base craniotomy was performed. The mass could be easily visualized with gentle depression of the temporal lobe, and it appeared to be tan and pink in color. It had a dural attachment along the sylvian fissure, and it was removed en bloc once it was separated from the dura. During the process, there was yellow cystic content from the mass.

**Figure 3:**
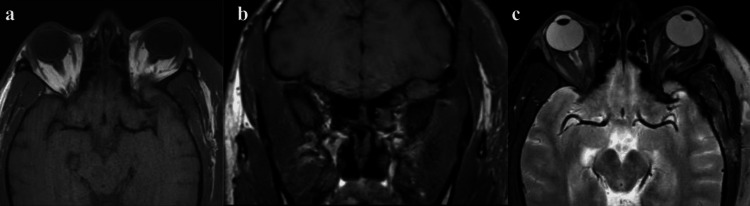
Postoperative MRI demonstrating no residual mass (a, b) Axial and coronal T1-weighted MR images showing no evidence for residual mass in the greater wing of the left sphenoid bone status post resection. (c) Axial T2-weighted MR image status post resection with no residual signal changes.

The patient recovered well, denying diplopia and endorsing slight improvement in proptosis. He was discharged on postoperative day four. Postoperative MRI showed gross total resection without residual tumor (Figure 3).

**Figure 4:**
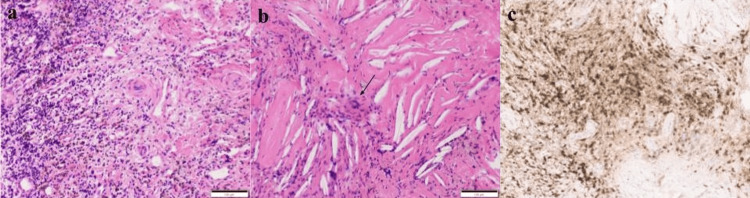
Pathological examination of the left sphenoid wing lesion Figure 4 depicts a pathological examination revealing a cystic lesion with attenuated epithelium lining, stromal fibrosis, hemosiderin, and associated inflammatory process (a) along with changes associated with a ruptured cyst, including a xanthogranulomatous reaction with cholesterol clefts and multinucleated giant cells (b, arrow). Immunohistochemical stains confirmed the CD3-positive T and CD20-positive B lymphocytic infiltrates (c).

Pathological examination revealed a cystic lesion with attenuated epithelium lining, stromal fibrosis, hemosiderin, and an associated inflammatory process (Figure 4a). Changes associated with a ruptured cyst were seen, including a xanthogranulomatous reaction with cholesterol clefts and multinucleated giant cells (Figure 4b, arrow). Immunohistochemical stains confirmed the CD3-positive T (Figure 4c) and CD20-positive B lymphocytic infiltrates, whereas the Gram and Grocott methenamine silver (GMS) fungal stains were negative (not shown), consistent with the final diagnosis of a ruptured cholesteatoma.

## Discussion

Cholesteatoma is a benign lesion with a squamous epithelium capsule and keratin debris, which gives it a pearly appearance [1]. Intracranial cholesteatoma is a rare entity that mostly occurs in the middle ear or mastoid region due to chronic otitis media or iatrogenic tympanostomy [2]. There was a case report of a large congenital petrous apex cholesteatoma invading the sphenoid, temporal, and occipital bone requiring radical mastoidectomy and transsphenoidal marsupialization and routine office debridement [6]. Another rare case of sellar cholesteatoma was thought to be due to the proliferation of squamous cell crests from the anterior hypophysis [7]. Additionally, there are reported cases of sphenoid wing epidermoid cysts, and the authors postulated that mislocated epithelial nests during the embryologic fusions of the sphenoid wing later on lead to the development of epidermoid cysts [5,8].

Although they are benign lesions, cholesteatomas can cause local destruction and present with Gradenigo-like symptoms, such as in the case of petrous apex cholesteatoma, or hypopituitarism, such as in the case of sellar cholesteatoma [6,7]. In our case, the patient presented with progressive proptosis and diplopia due to worsening mass effect upon the lateral orbital content. This presentation should raise concerns and prompt clinicians to pursue imaging studies. CT is the imaging of choice to evaluate the bony erosions, especially in cases of middle ear or mastoid cholesteatoma [1,4,5]. However, MRI is necessary to differentiate between cholesteatoma, a relatively benign lesion, and cerebral abscess, sometimes a neurosurgical emergency [2-4]. On rare occasions, typically with incomplete initial excision, cholesteatomas can undergo malignant transformation, which carries a poor prognosis [2,5]. Our patient had a unique presentation, with no clear history of trauma; however, a possible risk factor was an HIV-positive status, which has been recognized as a risk factor in developing cholesteatomas and possibly contributing to a more aggressive nature [9]. Given its unclear etiology, rate of growth, and mass effect, resection of this cholesteatoma was a reasonable treatment.

## Conclusions

Our report is limited by a single case study and no long-term follow-up besides immediate postoperative imaging. Due to limited studies in the literature, there is no clear size or rate of growth as a guideline for resection. Overall, we recommend that, upon discovery with workup, sphenoid wing cholesteatomas should be followed with serial imaging, initially an MRI at six-month intervals, and resection may be offered when mass effect or rapid interval growth is present.
